# Association between maternal blood or cord blood metal concentrations and catch-up growth in children born small for gestational age: an analysis by the Japan environment and children’s study

**DOI:** 10.1186/s12940-024-01061-7

**Published:** 2024-02-10

**Authors:** Tomozumi Takatani, Rieko Takatani, Akifumi Eguchi, Midori Yamamoto, Kenichi Sakurai, Yu Taniguchi, Yayoi Kobayashi, Chisato Mori, Michihiro Kamijima, the Japan Environment and Children’s Study Group

**Affiliations:** 1 Department of Pediatrics, Graduate School of Medicine, Chiba University, 1-8-1 Inohana, Chuo-ku, Chiba, 260-8670 Japan; 2 Center for Preventive Medical Sciences, Chiba University, Chiba, 263-8522 Japan; 3 Centre for Health and Environmental Risk Research, National Institute for Environmental Studies, Ibaraki, 305- 8506 Japan; 4 Department of Bioenvironmental Medicine, Graduate School of Medicine, Chiba University, Chiba, 260-8670 Japan; 5 Department of Occupational and Environmental Health, Nagoya City University Graduate School of Medical Sciences, 1 Kawasumi, Mizuho-cho, Mizuho-ku, Nagoya, 467-8601 Japan

**Keywords:** Small for gestational age, Heavy metal, Catch-up growth, Prenatal period

## Abstract

**Background:**

Catch-up growth issues among children born small for gestational age (SGA) present a substantial public health challenge. Prenatal exposure to heavy metals can cause adverse effects on birth weight. However, comprehensive studies on the accurate assessment of individual blood concentrations of heavy metals and their effect on the failure to achieve catch-up growth remain unavailable. This study aimed to evaluate the effects of uterine exposure to toxic metals cadmium, lead, and mercury and essential trace metals manganese and selenium at low concentrations on the postnatal growth of children born SGA.

**Methods:**

Data on newborn birth size and other factors were obtained from the medical record transcripts and self-administered questionnaires of participants in the Japan Environment and Children’s Study. The blood concentrations of lead, cadmium, mercury, selenium, and manganese in pregnant women in their second or third trimester were determined by inductively coupled plasma mass spectrometry. These heavy metal concentrations were also assessed in pregnant women’s cord blood. Furthermore, the relationship between each heavy metal and height measure/catch-up growth in SGA children aged 4 years was analyzed using linear and logistic regression methods. These models were adjusted for confounders.

**Results:**

We studied 4683 mother–child pairings from 103,060 pregnancies included in the Japan Environment and Children’s Study. Of these, 278 pairs were also analyzed using cord blood. At 3 and 4 years old, 10.7% and 9.0% of children who were born below the 10th percentile of body weight had height standard deviation scores (SDSs) below 2, respectively. Cord blood cadmium concentrations were associated with the inability to catch up in growth by 3 or 4 years old and the height SDS at 3 years old. In maternal blood, only manganese was positively associated with the height SDS of SGA children aged 2 years; however, it was not significantly associated with catch-up growth in these children.

**Conclusion:**

Cadmium exposure is associated with failed catch-up development in SGA children. These new findings could help identify children highly at risk of failing to catch up in growth, and could motivate the elimination of heavy metal (especially cadmium) pollution to improve SGA children’s growth.

**Supplementary Information:**

The online version contains supplementary material available at 10.1186/s12940-024-01061-7.

## Introduction

Children whose birth weight falls below the 10th percentile are classified as small for gestational age (SGA). Although reduced growth underlying SGA has multiple etiologies (including genetic factors), environmental factors greatly influence SGA pathogenesis. In fact, postnatal environmental changes enable 85–90% of SGA children to have spontaneous catch-up development during the first 2 years [1–3]. However, the remaining percentage cannot catch up from 2 to 4 years of age [4, 5]. Data from cohort studies have shown that catch-up growth failure affects later life outcomes, such as academic performance and adult earnings [6].

Several studies have highlighted concerns regarding the effects of exposure to hazardous metals, such as lead (Pb), cadmium (Cd), and mercury (Hg), as well as necessary trace elements, on child health [7–10]. Exposure to metals in the prenatal period can affect prenatal growth, potentially leading to SGA; thus, heavy metals could be major environmental determinants of birth outcomes [11–13]. Moreover, intrauterine exposure to heavy metals is associated with childhood neurobehavioral problems, such as attention-deficit/hyperactivity disorder [14, 15]. However, the effect of prenatal exposure to heavy metals on catch-up growth remains insufficiently understood. Hence, a large sample of children born SGA needs to be studied. A substantial sample size derived from a suitable reference population is required to obtain accurate and reliable data, considering that the criteria for defining SGA include a birth weight below the 10th percentile.

The heavy metals Pb, Cd, and Hg have detrimental effects on the health and developmental processes of children [16]. Manganese (Mn) is a vital trace element that is crucial for maintaining human health. Nevertheless, a study showed that exposure to Mn can have a neurotoxic effect on young children [17]. Selenium (Se) is also essential for maintaining human health. The regulatory capacity of Se can mitigate the adverse effects of Hg [18–21]. However, the optimal concentration range for Se is limited, and excessive doses can result in deleterious consequences [22]. The metals selected for evaluating the effects of heavy metal exposure in our study were determined according to the available data [23].

This study aimed to evaluate the effects of uterine exposure to toxic metals Cd, Pb, and Hg and essential trace metals Mn and Se at low concentrations on the postnatal growth of children born SGA.

## Materials and methods

### Study population

**Fig. 1:**
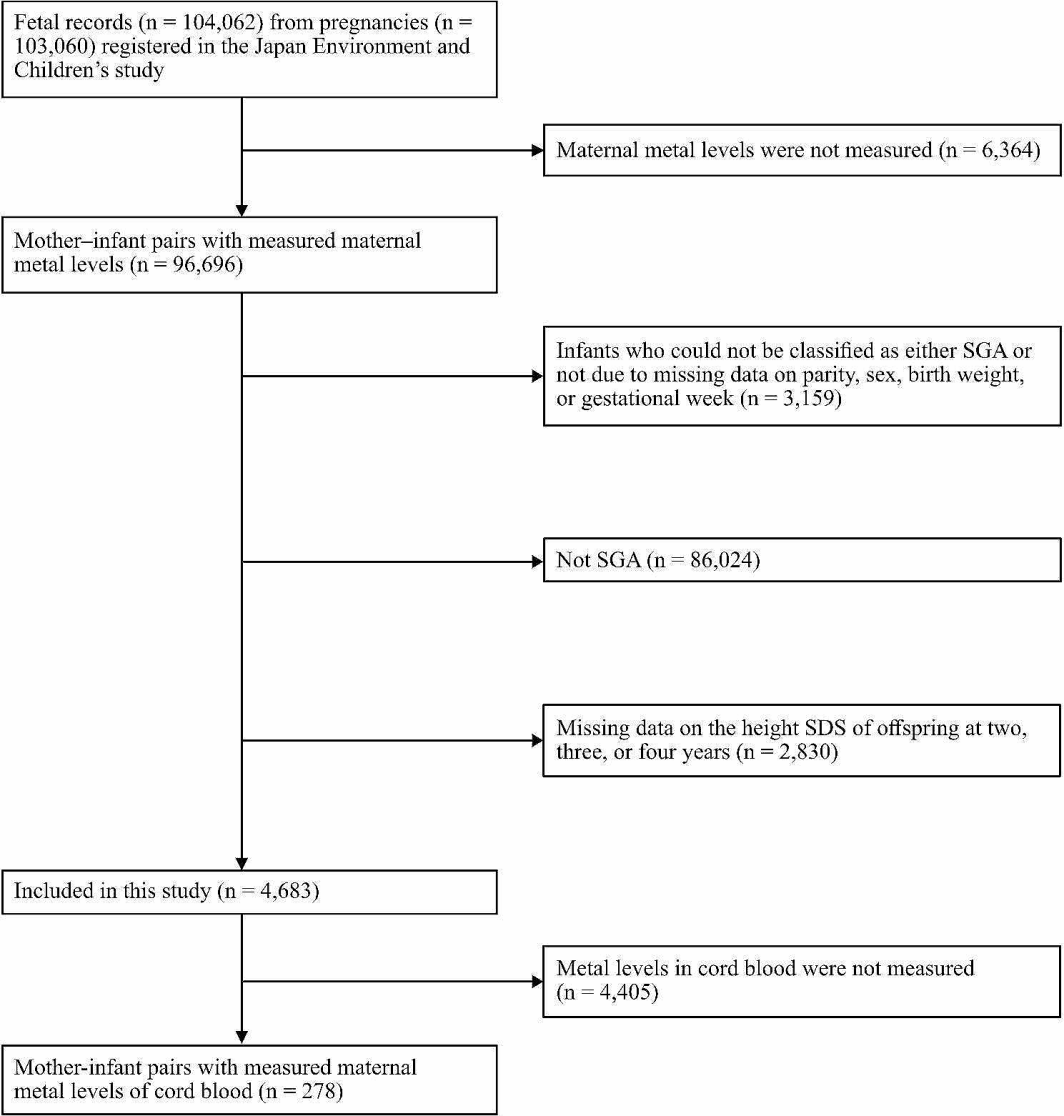
Participants’ flowchart. Blood heavy metal concentrations were measured in 96,693 expectant women with 103,060 pregnancies who were registered in the Japan Environment and Children’s Study. This study excluded children whose small-for-gestational-age (SGA) status could not be determined because of the lack of data on parity, sex, birth weight, or gestational age. Children who were not SGA and had missing height standard deviation score (SDS) data at 2, 3, at 4 years were also excluded. Finally, 4683 individuals were analyzed. Those with available information on metal concentrations in cord blood were also analyzed as a subcohort

The Japan Environment and Children’s Study (JECS), which is a nationwide prospective study, provided the data for this research. These data included 104,062 fetal records from 103,060 pregnancies. They were collected from a nationwide cohort that represented the general population. The cohort also comprised children born SGA. The JECS methodology [24] was registered as an observational cohort study in the University Hospital Medical Information Network Clinical Trials Registry (UMIN000030786). From January 2011 to March 2014, the JECS recruited women in their early stages of pregnancy across various residential areas in Japan, with 15 regional centers established. The inclusion criteria were as follows: residency in the study areas; expected delivery date between August 1, 2011, and mid-2014; continuous residence in Japan for the foreseeable future; and the ability to comprehend Japanese and complete a self-administered questionnaire. Those residing outside the study areas were excluded, even if they visit cooperating healthcare providers within those areas. As a subcohort study of the JECS, metal concentrations in cord blood were measured from randomly sampled participants who had comprehensive questionnaire data, medical record transcripts, and biospecimens obtained from the pregnancy period until the infants turned 6 months old [25]. In 103,060 pregnancies registered in the JECS, blood samples from 96,696 pregnant women were used to measure Cd, Hg, Pb, Se, and Mn concentrations. In every sample, the concentrations of all five metals were measured. We excluded participants who had at least one missing data point of variables used to determine the percentile of birth weight; these variables included sex, parity, birth weight, and gestational weeks. Next, we excluded 86,024 newborns with birth weights that exceeded the 10th percentile. Participants with at least one missing data point of height measurements at 2, 3, or 4 years of age were also excluded. Finally, this study included 4,683 mothers and their children for the analysis (Fig. 1). The JECS protocol was comprehensively reviewed and subsequently approved by the Institutional Review Board on Epidemiological Studies of the Ministry of the Environment, as well as the ethics committees of all participating institutions. This study followed the guidelines set forth in the Declaration of Helsinki and its subsequent revisions.

### Determination of metal concentrations

Metal concentrations in pregnant women were assessed as previously described (Nakayama et al., 2019). In the second or third trimester, 33 ml of blood sample was drawn from the peripheral vein of each participant during a medical visit. The blood samples were then partitioned into tubes with a volume of 3 ml, each containing sodium ethylenediaminetetraacetic acid, to analyze metal concentrations. Thereafter, these samples were transported to a central laboratory within 48 h and subsequently stored at −80 °C until measurements were performed. Metal concentrations in the blood samples were evaluated using an Agilent 7700 inductively coupled plasma mass spectrometer equipped with an autosampler (Agilent Technologies, Tokyo, Japan). Nakayama et al. (2019) found that the Mn, Se, Pb, Cd, and Hg concentrations in whole blood surpassed their respective detection thresholds, which were determined to be 0.522, 0.837, 0.129, 0.0234, and 0.049 ng/g, respectively.

### Variables

Data on the weight, length, and covariates at birth were collected as previously described [26]. Briefly, from the medical records, the following data were extracted: parity, birth size, maternal age at delivery, child sex, gestational length, pregnancy complications (hypertensive disorders of pregnancy, diabetes mellitus, and gestational diabetes mellitus), and delivery mode. The abovementioned records were transcribed during the gestational period and in the postpartum phase. Furthermore, we conducted a self-administered survey during study enrollment and once more at the later stages of pregnancy to collect data on the annual household income, educational attainment, and smoking and alcohol use during the course of pregnancy. Using both medical record transcripts and questionnaires, we collected data on prepregnancy height and weight, and maternal age. The body mass index (BMI) was measured using the formula (weight)/(height)^2^. In addition, the postnatal body measurements of children were determined from questionnaires. Covariates were selected in advance according to biological plausibility and the literature [27–33].

### Statistics

Children with a birth weight below the 10th percentile for their gestational age, parity, and sex in the Japanese standard [34] were considered to be SGA. The birth weight percentile was measured using a clinical tool developed by the Japanese Society of Pediatric Endocrinology (2011); this tool is based on Microsoft Excel. Children’s growth was assessed using this tool. Height standard deviation scores (SDS) according to the growth standard charts for Japanese children using healthy population [35] were also calculated at respective age by this tool. Adjusted differences (β) and 95% confidence intervals (CIs) for the height SDS at 2, 3, and 4 years of age were identified using multiple linear regression models. The distribution of Pb, Hg, Cd, Mn, and Se concentration data was skewed. Therefore, the log2-transformed values of these metals were used in the models. The associations between metal concentrations in maternal blood or cord blood and the inability of SGA children to achieve a height SDS above − 2 SDs at 2, 3, or 4 years of age were also analyzed using the multiple logistic regression models. Achieving catch-up growth meant having achieved a height of more than − 2 SDs, in accordance with a previous report [36]. Furthermore, adjusted odds ratios (ORs) and their corresponding 95% CIs were calculated to assess the associations between the failure to attain a height above −2 SDs and changes in metal concentrations measured on a log2 scale. The statistical models were adjusted for the following factors: maternal age (< 25, 25–29, 30–34, and ≥ 35 years), prepregnancy BMI (< 18.5, 18.5–<25, and ≥ 25.0 kg/m^2^), maternal education (< 10, 10–12, 13–16, and ≥ 17 years of education), annual household income (< 2, 2–<4, 4–<6, 6–<8, 8–<10, and ≥ 10 million Japanese yen), smoking status (never smoked, stopped smoking before pregnancy, and those who smoked during pregnancy), alcohol consumption (never drank, stopped drinking before pregnancy, and drank during pregnancy), hypertensive disorders of pregnancy (yes or no), gestational diabetes mellitus (yes or no), and diabetes mellitus (yes or no). Multiple imputations by chained equations (MICE) were used in the linear and logistic regression models to account for missing data in several covariates including prepregnancy maternal BMI, maternal education, annual household income, smoking status, and alcohol consumption (missing data rates: 0.02%, 0.9%, 7.2%, 1.0%, and 0.9%, respectively). This methodological approach was used to mitigate potential selection bias. All model variables were included in the imputation model, and linear and logistic regression analyses were performed in 50 imputed datasets, and the pooled estimates were reported. All statistical data were analyzed using R version 4.0.3 (R Development Core Team, Vienna, Austria; http://www.R-project.org).

## Results

### Study population

Tables 1 and 2 list the participants’ characteristics. The mean age at delivery was 31.3 years (SD = 5.0). Approximately 90% of the participants showed a prepregnancy BMI below 25 kg/m^2^. The mean length of gestation was 39.0 weeks. In addition, 10.7% and 9.0% of children born with a birth weight below the 10th percentile and showed a height below −2 SDs at 3 and 4 years of age, respectively. All characteristics, except for alcohol consumption, showed significant differences among all pregnancies. Supplementary Table 1 shows the mean, SD, quartile concentrations, and ranges of metal exposure in maternal blood. Those of metal concentrations in cord blood samples are shown in Supplementary Table 2.

**Table 1:** Maternal characteristics 6691282165####

Characteristics	Current study (*n* = 4,683)	All pregnancies (*n* = 103,060)	
	n (%)	n (%)	p-value*
Age at delivery (years), mean ± SD	31.9 ± 4.8	31.2 ± 5.1	< 0.01
<25	295 (6.3)	10,125 (9.8)	
25–<30	1,206 (25.8)	27,760 (26.9)	
30–<35	1,732 (37.0)	35,650 (34.6)	
≥35	1,450 (31.0)	27,414 (26.6)	
Missing	0 (0.0)	2,111 (2.0)	
Education (years)			< 0.01
<10	144 (3.1)	4,726 (4.6)	
10–<13	2,558 (54.6)	54,579 (53)	
13–<17	1,867 (39.9)	36,740 (35.6)	
≥17	74 (1.6)	1,434 (1.4)	
Missing	40 (0.9)	5,581 (5.4)	
Parity			< 0.01
0	2,080 (44.4)	40,395 (39.2)	
≥1	2,603 (55.6)	59,642 (57.9)	
Missing	0 (0.0)	3,023 (2.9)	
Prepregnancy BMI (kg/m^2^), mean ± SD	20.5 ± 2.9	21.2 ± 3.3	< 0.01
<25	1,082 (23.1)	16,629 (16.1)	
25–<30	3,271 (69.8)	74,816 (72.6)	
≥30	329 (7.0)	10,939 (10.6)	
Missing	1 (0.0)	676 (0.7)	
Annual household income (million Japanese yen)			< 0.01
<2	210 (4.5)	5,167 (5.0)	
2–<4	1,425 (30.4)	31,460 (30.5)	
4–<6	1,453 (31.0)	30,082 (29.2)	
6–<8	750 (16.0)	14,486 (14.1)	
8–<10	302 (6.4)	5,954 (5.8)	
≥10	207 (4.4)	3,888 (3.8)	
Missing	336 (7.2)	12,023 (11.7)	
Smoking status			< 0.01
Never smoked	2,897 (61.9)	56,156 (54.5)	
Quit before pregnancy	1,008 (21.5)	23,280 (22.6)	
Smoked in early pregnancy	732 (15.6)	17,791 (17.3)	
Missing	46 (1.0)	5,833 (5.7)	
Alcohol consumption			0.26
Never consumed	1,607 (34.3)	32,615 (31.6)	
Stopped consuming before pregnancy	770 (16.4)	16,740 (16.2)	
Consumed in early pregnancy	2,264 (48.3)	47,865 (46.4)	
Missing	42 (0.9)	5,840 (5.7)	
Hypertensive disorders of pregnancy	348 (7.4)	3,145 (3.1)	< 0.01
Missing	0 (0.0)	2,375 (2.3)	
Gestational diabetes mellitus	48 (1.0)	2,713 (2.6)	< 0.01
Missing	0 (0.0)	2,375 (2.3)	
Diabetes mellitus	133 (2.8)	1,101 (1.1)	< 0.01
Missing	0 (0.0)	2,375 (2.3)	

Note: BMI: Body mass index; SD: Standard deviation

* Chi-square test was performed for each characteristic

**Table 2:** Infant characteristics

	All (*n* = 4,683)
	n (%)
Sex	
Male	2,371 (50.6)
Female	2,312 (49.4)
Mode of Delivery	
Vaginal	3,500 (74.7)
Cesarean	1,169 (25.0)
Missing	14 (0.3)
Birth weight (g), mean ± SD	2,383.7 ± 349.8
Gestational week, mean ± SD	39.0 ± 2.0
Height SDS < −2 at 2 years of age	619 (13.2)
Height SDS < −2 at 3 years of age	502 (10.7)
Height SDS < −2 at 4 years of age	422 (9.0)

Note: SD: Standard deviation; SDS: Standard deviation score;

SGA: Small for gestational age

### Associations between maternal metal concentrations, birth size, and SGA

Table 3 presents the associations between maternal metal concentrations and the height SDS of offspring at 2, 3, or 4 years of age. The analysis comprised 4683 mother–child pairs. To account for any missing data in the covariates, we employed MICE. In the linear regression analysis, only Mn was associated with the height SDS of children born SGA at 2 years of age. No other maternal heavy metal concentrations were associated with the height SDS in children born SGA at 2, 3, or 4 years of age (Table 3). In the logistic regression analysis, no heavy metal concentrations in maternal blood were associated with the failure to achieve children’s height SDS above −2 at 3 or 4 years of age (Table 4). However, in cord blood, Cd concentrations were associated with the height SDS in SGA children at 3 years old (β = −0.29, 95% CI: −0.55, −0.04) (Table 5). Moreover, high Cd concentrations in cord blood increased the odds of failure to achieve children’s height SDS above − 2 at 3 or 4 years of age (OR = 2.35; 95% CI: 1.02, 5.43 and OR = 2.60, 95% CI: 1.06, 6.40, respectively) (Table 6).

**Table 3:** Regression coefficients for blood metal concentrations in relation to measures of height SDS

Participants	Metal	Height SDS at 2 years of age	Height SDS at 3 years of age	Height SDS at 4 years of age
		β (95% CI)	β (95% CI)	β (95% CI)
*N* = 4,683	Mn	0.42 (0.06, 0.79)	0.05 (−0.04, 0.14)	0.02 (−0.07, 0.12)
	Pb	0.06 (−0.24, 0.37)	−0.03 (−0.10, 0.05)	−0.07 (−0.15, 0.01)
	Se	0.05 (−0.90, 0.99)	−0.08 (−0.31, 0.16)	−0.12 (−0.36, 0.12)
	Hg	0.12 (−0.09, 0.33)	0.02 (−0.04, 0.07)	0.02 (−0.03, 0.08)
	Cd	0.05 (−0.20, 0.29)	0.04 (−0.02, 0.10)	0.05 (−0.01, 0.11)

Note: Models were adjusted for maternal age, prepregnancy body mass index, alcohol consumption and smoking status, hypertensive disorders of pregnancy, gestational diabetes mellitus, diabetes mellitus, income, and education

Log2-transformed metal concentrations were used for the models

Cd: Cadmium; Hg: Mercury; Mn: Manganese; Pb: Lead; Se: Selenium; CI: Confidence interval; SGA: Small for gestational age; SDS: Standard deviation score

**Table 4:** Odds ratios for blood metal concentrations in relation to catch-up growth failure

Participants	Metal	Height SDS < −2 at 2 years of age	Height SDS < −2 at 3 years of age	Height SDS < −2 at 4 years of age
		OR (95% CI)	OR (95% CI)	OR (95% CI)
*N* = 4,683	Mn	0.89 (0.72, 1.09)	0.97 (0.77, 1.21)	0.83 (0.65, 1.05)
	Pb	1.11 (0.94, 1.32)	1.01 (0.83, 1.22)	1.09 (0.89, 1.34)
	Se	0.94 (0.55, 1.60)	1.12 (0.62, 2.00)	1.30 (0.69, 2.44)
	Hg	1.00 (0.89, 1.12)	0.99 (0.87, 1.12)	0.97 (0.84, 1.10)
	Cd	0.98 (0.85, 1.12)	0.97 (0.83, 1.13)	0.94 (0.80, 1.11)

Note: Models were adjusted for maternal age, prepregnancy body mass index, alcohol consumption and smoking status, hypertensive disorders of pregnancy, gestational diabetes mellitus, diabetes mellitus, income, and education

Log2-transformed metal concentrations were used for the models

Cd: Cadmium; Hg: Mercury; Mn: Manganese; Pb: Lead; Se: Selenium; CI: Confidence interval; OR: Odds ratio; SGA: Small for gestational age; SDS: Standard deviation score

**Table 5:** Regression coefficients for cord blood metal concentrations in relation to height SDS at respective age

Participants	Metal	Height SDS at 2 years of age	Height SDS at 3 years of age	Height SDS at 4 years of age
		β (95% CI)	β (95% CI)	β (95% CI)
*N* = 278	Mn	−0.03(−0.55, 0.49)	−0.15 (−0.43, 0.14)	−0.03 (−0.56, 0.51)
	Pb	−0.17 (−0.57, 0.24)	−0.10 (−0.32, 0.12)	−0.25 (−0.67, 0.17)
	Se	0.54 (−0.63, 1.70)	0.03 (−0.61, 0.67)	0.59 (−0.62, 1.79)
	Hg	−0.05 (−0.33, 0.23)	0.08 (−0.08, 0.23)	−0.14 (−0.44, 0.15)
	Cd	−0.45 (−0.90, 0.01)	−0.29 (−0.55, −0.04)	−0.12 (−0.59, 0.35)

Note: Models were adjusted for maternal age, prepregnancy body mass index, alcohol consumption and smoking status, hypertensive disorders of pregnancy, gestational diabetes mellitus, diabetes mellitus, income, and education

Log2-transformed metal concentrations were used for the models

Cd: Cadmium; Hg: Mercury; Mn: Manganese; Pb: Lead; Se: Selenium; CI: Confidence interval; SGA: Small for gestational age; SDS: Standard deviation score

**Table 6:** Odds ratios for blood metal concentrations in relation to catch-up growth failure

Participants	Metal	Height SDS < −2 at 2 years of age	Height SDS < −2 at 3 years of age	Height SDS < −2 at 4 years of age
		OR (95% CI)	OR (95% CI)	OR (95% CI)
*N* = 278	Mn	0.87 (0.35, 2.18)	1.03 (0.38, 2.82)	0.88 (0.29, 2.71)
	Pb	1.50 (0.72, 3.11)	1.49 (0.67, 3.32)	1.19 (0.52, 2.74)
	Se	1.00 (0.13, 7.64)	0.87 (0.10, 7.88)	0.65 (0.06, 7.12)
	Hg	0.81 (0.49, 1.37)	0.88 (0.50, 1.54)	0.81 (0.44, 1.47)
	Cd	1.74 (0.81, 3.74)	2.35 (1.02, 5.43)	2.60 (1.06, 6.40)

Note: Models were adjusted for maternal age, prepregnancy body mass index, alcohol consumption and smoking status, hypertensive disorders of pregnancy, gestational diabetes mellitus, diabetes mellitus, income, and education

Log2-transformed metal concentrations were used for the models

Cd: Cadmium; Hg: Mercury; Mn: Manganese; Pb: Lead; Se: Selenium; CI: Confidence interval; OR: Odds ratio; SGA: Small for gestational age; SDS: Standard deviation score

## Discussion

This study examined the effect of maternal heavy metal exposure on the catch-up growth of children born SGA. A previous report [36] using the same cohort as our study conveyed a different prevalence rate of the failure to achieve catch-up growth, possibly because they used a different definition. This past report used the clinical definition of SGA for growth hormone treatment. In our study, the rate of failure to achieve a height SDS above − 2 among SGA offspring was approximately 10% at 3 or 4 years of age, consistent with the findings of previous studies [3, 37]. Generally, infantile growth retardation can be caused by environmental factors *in utero*, including low perfusion in the placenta resulting from maternal hypertension or inborn errors, such as chromosomal abnormalities. If growth retardation is caused by the intrauterine environment, children may exhibit catch-up growth when exposed to an improved environment following birth. However, some of these children fail to achieve catch-up growth, requiring growth hormone therapy. The concentrations of metals such as Pb, Cd, Se, Hg, and Mn in mothers were previously reported to be associated with SGA occurrence [13]. However, the effect of these metals on child growth is still unclear. In the present study, the Mn concentration in maternal blood had a slightly but significantly positive effect on the children’s height SDS at 2 years of age. Mn is indispensable for maintaining certain organ functions and is incorporated into several enzymes crucial for cellular defense against oxidative stress, including Mn superoxide dismutase, which promotes cell function and growth. Conversely, cord blood Mn showed no positive effect on postnatal growth, suggesting that Mn can modify placental function and impact postnatal growth. Placental weight reportedly positively correlates with postnatal growth [38]. Additionally, maternal blood concentration, rather than cord blood concentration, has been reported to be significantly associated with fetal growth, likely through placental function [39, 40]. Moreover, Cd concentrations in cord blood were associated with the achievement of a height SDS above − 2 at 3 and 4 years of age. According to several previous studies and our previous study, prenatal Cd exposure is associated with birth height and weight [13, 41, 42]. Cd exposure was also related to growth until the age of 3–5 years [43–45], consistent with our findings. In the present study, the average Cd concentration observed in cord blood was approximately 6% of that found in maternal blood. Thus, while the placenta acts as a major barrier against Cd, it remains insufficient in fully preventing Cd transfer. This finding is consistent with that in previous studies [41, 44]. In fact, a significant correlation was observed between Cd in maternal blood and Cd in cord blood (*r* = 0.58, *p* < 0.01, shown in Supplementary Fig. 1), although this correlation is not perfect. Placental Cd transfer, which varies among individuals, may contribute to the inconsistency in results in the association between Cd in maternal blood or cord blood and the postnatal growth of SGA children. Further research is needed. In any case, cord blood was a better biomarker than maternal blood in predicting growth at 3 and 4 years of age among SGA children. Smoking is a recognized source of Cd [46, 47]. In our study population, 15.6% reported smoking in early pregnancy. Despite adjusting for smoking status in our multiple regression and logistic analyses, Cd exposure remained statistically significant. Food might be the main source of Cd exposure of pregnant women in our cohort. Another study using the JECS cohort showed that Cd in Japanese pregnant women was mainly obtained from the diet and not from the soil, house dust, or indoor air [48]. Once consumed, Cd stays mainly in the kidneys and liver, where it can affect the human body for a long time, considering that its half-life is 10–30 years [49]. Caspase-3, caspase-9, p53, and Fas are also affected by Cd exposure, leading to cellular apoptosis [50, 51]. Moreover, Cd induces oxidative stress and cellular growth impairment [52, 53]. Several in vitro studies have also shown that Cd exerts an effect on DNA methylation [54, 55] and can alter long-term cellular function via epigenetic effects. In fact, a human study using mother–newborn pairs suggested that Cd alters the methylation status in promoter regions across the whole genome [56]. Regarding epigenetic effects, researchers were focused on investigating the mechanism of the association between prenatal exposure and outcomes in later life [57, 58], and Cd might be involved in this process. Through the abovementioned mechanisms, prenatal Cd exposure may impair catch-up growth in SGA offspring. Further research on Cd including epigenetic analyses is warranted.

This study has several strengths. First, it used a large prospective cohort covering most of Japan; thus, our sample size is substantial despite the low number of births categorized as SGA according to the SGA definition. Second, our data were obtained through the direct analysis of maternal blood samples during the second or third trimester of pregnancy and cord blood samples. Third, we assessed catch-up growth over 4 years after birth in a substantial sample size of SGA births.

Our study has several limitations. First, self-administered questionnaires were used to gather information on multiple variables, such as smoking, alcohol use, maternal education, and family income; hence, recall or social desirability bias may have been introduced. Second, this study could not account for all confounding variables, including maternal nutritional status and exposure to other environmental contaminants, which can affect maternal metal concentration and postnatal growth in children. Third, this study analyzed both samples collected at the second and third trimesters. Different time points may affect the analysis. Fourth, given that our study only analyzed mothers with children born SGA, many JECS participants were excluded. Mothers with SGA infants may also differ from the general population, so caution is necessary when considering the general population. Moreover, 2830 participants were excluded because of missing outcome data, indicating a potential selection bias.

## Conclusions

Mn in maternal blood is positively associated with SGA children’s height at 2 years. Moreover, increased prenatal Cd exposure is associated with catch-up growth failure in SGA children by the ages of 3 and 4 years. Cd concentrations in cord blood could be a better indicator for catch-up growth failure than those in maternal blood. While further research is required to determine the mechanism of Cd’s effect, a reduction in heavy metal (especially Cd) exposure in pregnant women is necessary to improve catch-up growth in SGA children.

### Electronic supplementary material

Below is the link to the electronic supplementary material.

Supplementary Material 1

Supplementary Material 2

Supplementary Figure 3. Scatter plots of cadmium in maternal blood and cord blood. Scatter plots show that cadmium (Cd) in maternal blood has a moderate correlation with Cd in cord blood (R = 0.54; p < 0.01)

## Data Availability

The data are unsuitable for public deposition owing to ethical restrictions and the legal framework of Japan.

## Declarations

### Data references

The datasets of jecs-ta-20190930 and jec-qa-20210401 were used for this analysis.

## Abbreviations

BMI: Body mass index
CI: Confidence interval
DM: Diabetes mellitus
GDM: Gestational diabetes mellitus
JECS: Japan Environment and Children’s Study
MICE: Multiple imputations by chained equations
OR: Odds ratio
HDP: Hypertensive disorders of pregnancy
SD: Standard deviation
SDS: Standard deviation score
SGA: Small for gestational age
Cd: Cadmium
Hg: Mercury
Pb: Lead
Mn: Manganese
Se: Selenium
