# Supplementary material for: Association between maternal blood or cord blood metal concentrations and catch-up growth in children born small for gestational age: an analysis by the Japan environment and children’s study
Source: Environ Health. 2024 Feb 10;23:18. doi: 10.1186/s12940-024-01061-7 (PMC10858588; doi:10.1186/s12940-024-01061-7)
Supplement: Supplementary file 1 — Supplementary Material 1 [file 12940_2024_1061_MOESM1_ESM.docx]

| **Supplementary Table 1** |  |  |  |  |  |  |  |
| --- | --- | --- | --- | --- | --- | --- | --- |
| **Metal concentrations in maternal blood** | |  |  |  |  |  |  |
|  | Mean | SD | Minimum | 25^th^ Percentile | Median | 75^th^ Percentile | Maximum |
| Mn (ng/g) | 15.64 | 4.73 | 4.12 | 12.30 | 15.00 | 18.30 | 41.80 |
| Pb (ng/g) | 6.63 | 2.99 | 1.51 | 4.88 | 6.07 | 7.67 | 84.80 |
| Se (ng/g) | 171.10 | 20.50 | 110.00 | 157.00 | 169.00 | 183.00 | 322.00 |
| Hg (ng/g) | 4.35 | 2.64 | 0.34 | 2.63 | 3.73 | 5.32 | 30.40 |
| Cd (ng/g) | 0.78 | 0.41 | 0.14 | 0.50 | 0.68 | 0.93 | 4.68 |
| Note: Cd: Cadmium; Hg: Mercury; Mn: Manganese; Pb: Lead; Se: Selenium | | | |  |  |  |  |
