# Supplementary material for: Association between maternal blood or cord blood metal concentrations and catch-up growth in children born small for gestational age: an analysis by the Japan environment and children’s study
Source: Environ Health. 2024 Feb 10;23:18. doi: 10.1186/s12940-024-01061-7 (PMC10858588; doi:10.1186/s12940-024-01061-7)
Supplement: Supplementary file 2 — Supplementary Material 2 [file 12940_2024_1061_MOESM2_ESM.docx]

| **Supplementary Table 2** |  |  |  |  |  |  |  |
| --- | --- | --- | --- | --- | --- | --- | --- |
| **Metal concentrations in cord blood** | |  |  |  |  |  |  |
|  | Mean | SD | Minimum | 25^th^ Percentile | Median | 75^th^ Percentile | Maximum |
| Mn (ng/g) | 49.04 | 16.41 | 18.50 | 39.23 | 45.70 | 57.08 | 140.00 |
| Pb (ng/g) | 5.18 | 2.10 | 1.13 | 3.63 | 4.89 | 6.35 | 18.10 |
| Se (ng/g) | 179.60 | 24.59 | 119.00 | 164.00 | 179.00 | 194.80 | 270.00 |
| Hg (ng/g) | 8.93 | 5.68 | 1.15 | 5.62 | 7.70 | 10.68 | 47.90 |
| Cd (ng/g) | 0.05 | 0.02 | 0.02 | 0.03 | 0.05 | 0.06 | 0.19 |
| Note: Cd: Cadmium; Hg: Mercury; Mn: Manganese; Pb: Lead; Se: Selenium | | | |  |  |  |  |
