# Supplementary figures and images for: Association between maternal blood or cord blood metal concentrations and catch-up growth in children born small for gestational age: an analysis by the Japan environment and children’s study
Source: Environ Health. 2024 Feb 10;23:18. doi: 10.1186/s12940-024-01061-7 (PMC10858588; doi:10.1186/s12940-024-01061-7)

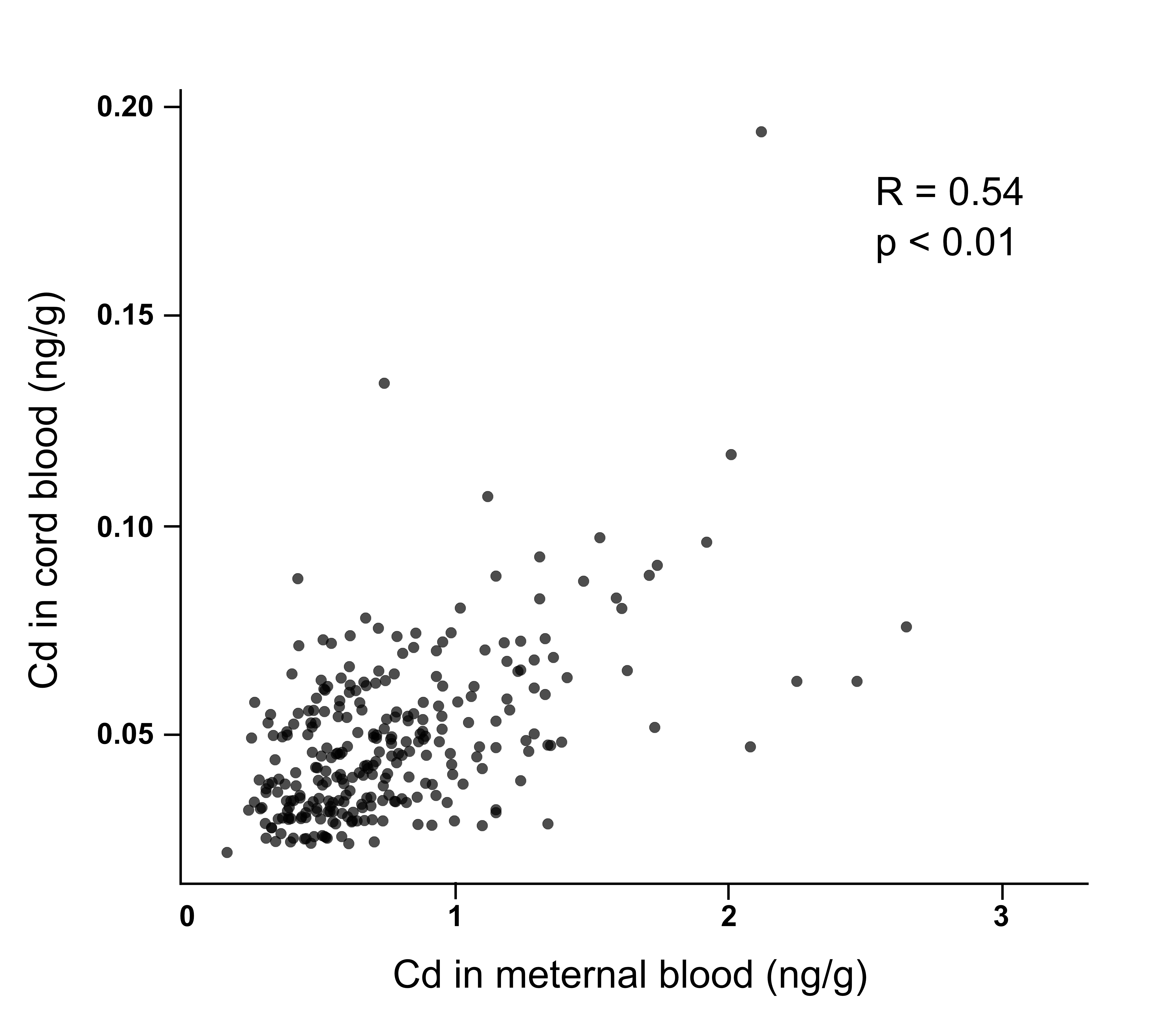

Supplement: Supplementary file 3 — Supplementary Figure 3. Scatter plots of cadmium in maternal blood and cord blood. Scatter plots show that cadmium (Cd) in maternal blood has a moderate correlation with Cd in cord blood (R = 0.54; p < 0.01) [file 12940_2024_1061_MOESM3_ESM.jpg]
